# Fluid dynamic lateral slicing of high tensile strength carbon nanotubes

**DOI:** 10.1038/srep22865

**Published:** 2016-03-11

**Authors:** Kasturi Vimalanathan, Jason R. Gascooke, Irene Suarez-Martinez, Nigel A. Marks, Harshita Kumari, Christopher J. Garvey, Jerry L. Atwood, Warren D. Lawrance, Colin L. Raston

**Affiliations:** 1 Flinders Centre for NanoScale Science & Technology, School of Chemical & Physical Sciences, Flinders University, Adelaide SA 5001, Australia; 2 Nanochemistry Research Institute, Department of Physics and Astronomy, School of Science, Curtin University, Bentley Campus, Perth, WA 6102, Australia; 3 Department of Chemistry, University of Missouri, 601 South College Avenue, Columbia, Missouri 65211, United States; 4 James L. Winkle College of Pharmacy, University of Cincinnati, 3225 Eden Avenue, Cincinnati, Ohio, 42567, United States; 5 Bragg Institute, Australian Nuclear Science and Technology Organisation, New Illawarra Road, Lucas Heights, 2234, NSW

## Abstract

Lateral slicing of micron length carbon nanotubes (CNTs) is effective on laser irradiation of the materials suspended within dynamic liquid thin films in a microfluidic vortex fluidic device (VFD). The method produces sliced CNTs with minimal defects in the absence of any chemical stabilizers, having broad length distributions centred at *ca* 190, 160 nm and 171 nm for single, double and multi walled CNTs respectively, as established using atomic force microscopy and supported by small angle neutron scattering solution data. Molecular dynamics simulations on a bent single walled carbon nanotube (SWCNT) with a radius of curvature of order 10 nm results in tearing across the tube upon heating, highlighting the role of shear forces which bend the tube forming strained bonds which are ruptured by the laser irradiation. CNT slicing occurs with the VFD operating in both the confined mode for a finite volume of liquid and continuous flow for scalability purposes.

The processing of cylindrical carbon nanotubes (CNTs) for exploiting their exceptional thermal, mechanical, and electrical properties^1^ poses a number of challenges. This includes overcoming a high degree of bundling and aggregation arising from their high aspect ratio and strong inter-tube van der Waals interactions. The CNTs are microns to millimetres in length, and may have single or multiple layers, described as single walled (SWCNTs), double walled (DWCNTs) or multi walled (MWCNTs). They have low density, and high stiffness and axial strength^2^ with an exceptionally high Young’s modulus, 1.0 to 1.28 TPa^3^,^4^,^5^. CNTs are produced using a number of techniques, including arc-discharge, laser ablation and chemical vapour deposition (CVD)^6^,^7^,^8^, typically affording arrays of nanotubes in various lengths. However, the availability of shorter lengths without compromising quality is important for many applications, for example, in incorporating them into lipid bilayers for molecular sensing^2^, and for enhancing the efficiency of electronic devices^3^,^4^,^9^, for solar cell applications^10^ and for chirality separation^11^,^12^,^13^. There has thus been considerable work directed at shortening CNTs towards sub-micron lengths, but this has proved challenging, currently requiring the use of high-energy sonication, lengthy processing times and the use of toxic chemicals^14^,^15^,^16^,^17^,^18^,^19^,^20^,^21^,^22^,^23^,^24^. Moreover, such conditions can chemically alter the surface of the CNTs with consequential change in their properties, thereby limiting their applications. Controlling the shortening of CNTs requires the use of a suitable processing medium in overcoming their tendency to aggregate, with some common solvents such as N-methyl-2-pyrollidone (NMP) and N,N-dimethylformamide (DMF) being effective dispersants for such purpose. The colloidal stability of individual CNTs usually requires polymer wrapping agents^25^ and surfactants^26^, and covalent end and/or sidewall functionalization^27^,^28^.

We have established a new means to achieve shortening of CNTs that avoids many of these problems. Despite their high tensile strength, CNTs can be laterally sliced in solution by applying intense shear within dynamic thin films in a vortex fluidic device (VFD) while irradiating at 1064 nm using a pulsed Q-switch Nd:YAG laser (Fig. 1a). The VFD microfluidic platform generates controllable mechanoenergy within the liquid medium, as thin films around the internal walls of a rapidly rotating tube, which for practical purposes was a 20 mm diameter (ID 16.000 ± 0.013 mm) borosilicate nuclear magnetic resonance (NMR) glass tube. In general, the optimal performance of the VFD occurs at high rotational speeds (2000 rpm to 9000 rpm) and inclination angles, θ > 0°, with a 45° tilt angle corresponding to the maximum cross vector of centrifugal force and gravity, being the optimum setting for a diverse number of applications of the device^29^,^30^,^31^,^32^,^33^,^34^,^35^.

## Results and Discussion

Both the confined and continuous modes of operation of the VFD are effective in slicing of the CNTs. In the confined mode a finite volume of liquid is wholly contained within the tube during processing while ensuring that a vortex is maintained to the bottom of the tube for moderate rotational speeds, and without any liquid exiting at the top of the tube. This minimises the formation of different shear regimes. In this mode, Stewartson/Ekman layers prevail in the resulting dynamic thin films, which arise from the liquid accelerating up the tube, with gravitational force acting against them^30^. In the continuous flow mode of operation of the VFD, jet feeds deliver one or more solutions (normally) to the bottom of the tube where there is intense micromixing, with instability of the liquid boundary layer in the hemispherical sphere at the bottom of the tube. The viscous drag as the liquid whirls up the tube creates shear, in addition to that from the Stewartson/Ekman layers. These unique fluidic properties of the VFD have led to a number of applications including control of chemical reactivity and selectivity in organic synthesis^29^,^30^, exfoliating graphene and boron nitride^31^, protein folding^32^, fabricating toroidal arrays of SWCNTs^33^, forming mesoporous silica at room temperature with control of pore size and wall thickness^34^, and probing the structure of self organised processes^35^,^36^.

**Figure 1:**
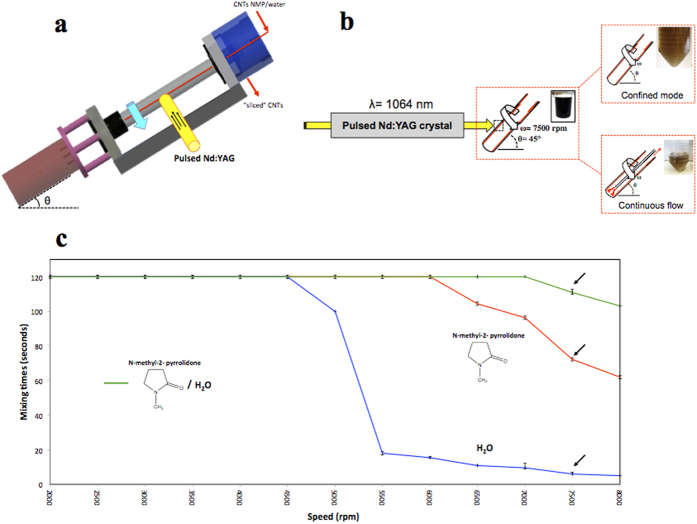
VFD laser processing and mixing times. (**a**) Schematic of the vortex fluidic device (VFD). (**b**) Experimental set up for laser Nd:YAG processing operating at 1064 nm wavelength, for confined and continuous flow modes of operation of the VFD. (**c**) Variation of mixing times of pure NMP, water and NMP/water at a 1:1 ratio. Mixing times were measured by placing 1 mL of the solvent in the VFD operating at an inclination angle of 45° and varying the rotational speed (2000–9000 rpm) and measuring the time taken for a drop of dye to uniformally mix with the bulk liquid (measured in triplicates).

The volume of liquid for confined mode studies was set at 1 mL with the speed and tilt angle varied to establish the lowest time for two liquids to homogenously mix. This was important to optimise exposure of the CNTs in the solution to the 8 mm diameter laser beam used in the ‘slicing’ experiments, as well as providing the maximum shear for debundling of the tubes. The minimum time taken for a homogenous mixture of water and NMP (1:1 ratio) to uniformly mix (visually estimated for solutions containing a dye, in the range 2000 to 9000 rpm, was for a rotational speed of 7500 rpm (Fig.1) for θ 45°. The choice of a 1:1 ratio of water and NMP was based on overcoming the low mixing time for NMP, in which the CNTs are readily dispersed, versus the fast mixing times in water, in which there is little dispersion of CNTs. These VFD parameters (θ 45^o^, 7500 rpm rotational speed) also correspond to the optimal processing parameters (*vide infra*).

Energy absorption from the pulsed laser source was essential to laterally slice the CNTs. The laser produces 1064 nm pulses of 5 ns duration and operates at a repetition rate of 10 Hz. In the confined mode, the optimised irradiation conditions were found to be a laser power of 260 mJ/pulse for, 30 minutes of processing. This was established by determining CNT length distributions using AFM on isolated samples (Supplementary Fig. S5). At high laser powers, ≥400 mJ, only small amounts of sliced nanotubes were evident, along with bundled and aggregated long nanotubes. Here the extra heat provided by the laser pulse disturbs the fluid flow, with a clear band which is presumably largely devoid of CNTs at the point of laser irradiation. Decreasing the laser power to <200 mJ for 30 minutes was ineffective in slicing the CNTs and no clear band was observed at the irradiation site. (Supplementary Fig. S5).

The optimal VFD processing parameters (inclination angle θ and rotational speed) were determined following a number of control experiments. For rotational speeds below 6000 rpm, the as-received aggregated bundles of CNTs (SWCNTs, DWCNTs and MWCNTs) remained unaffected in both the presence and absence of laser irradiation. At 6000 rpm and 45° tilt, a significant amount of debundling of the nanotubes occurred (as shown through AFM observations), but laser irradiation led to non-uniformity of the sliced CNTs, and the persistence of longer tubes. Uniform slicing of the CNTs occurred between 7500 rpm and 8000 rpm when irradiated by the pulsed laser. A tilt angle of 45° angle was found to be optimal, with minimal lateral slicing at other angles between 0 and 90°. For these optimised VFD processing parameters (θ 45°, rotational speed 7500 rpm), in the absence of the laser pulses there was no evidence for slicing, with the length of the CNTs unchanged, although there was significant debundling (Supplementary Fig. S1). In decoupling the effect of the VFD and the laser irradiation, a pulsed laser beam of the same optimised power was directed towards the CNTs in a 1:1 mixture of water and NMP in a glass cuvette rather than in a VFD. This resulted in fragmentation of the CNTs nanotubes into (i) large and irregular shapes for the SWCNTs, (ii) large bundles and aggregates for the DWCNTs, and (iii) irregular slicing of MWCNTs with bundles and aggregates present (Supplementary Fig. S2). In addition, there was no evidence of slicing the CNTs with the laser wavelength set to 532 nm (Supplementary Fig. S3). Thus, the optimised parameters for laterally slicing the different CNTs to lengths below *ca* 450 nm are established as a 45° tilt angle of the VFD tube rotating at 7500 rpm under confined mode for 30 minutes, with the 10 Hz pulsed laser operating at a wavelength of 1064 nm and 260 mJ per 5 ns pulse. These conditions were found to be independent of the concentration of CNTs in the solution up to a maximum concentration of 0.1 mg/mL, above which presumably the dynamics in the thin film are affected by the CNTs.

**Figure 2:**
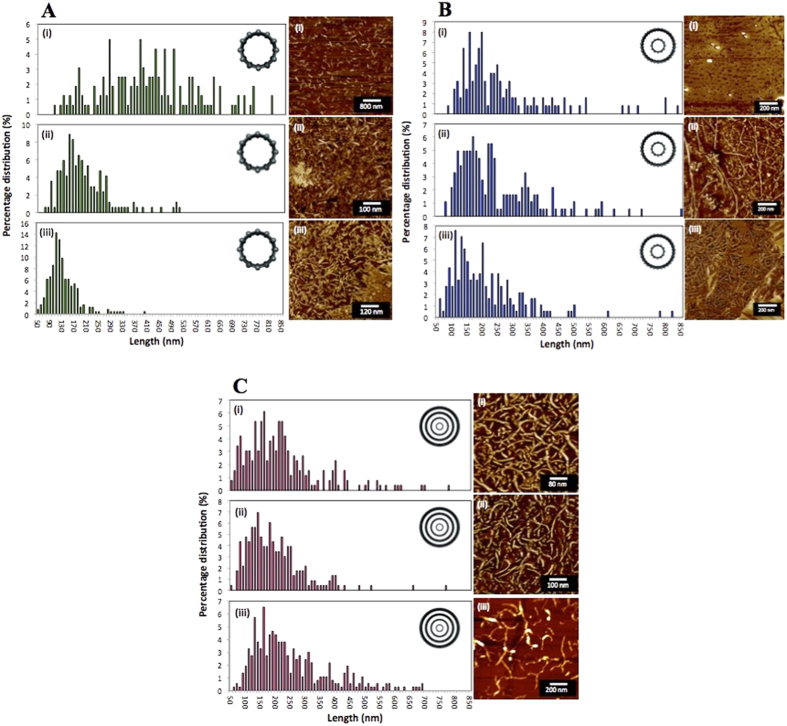
Slicing of SWCNTs, DWCNTs and MWCNTs at θ = 45° and rotational speed of 7500 rpm. AFM height images of laterally sliced CNTs using the confined mode of operation of the VFD, with associated length distribution plots for reaction times of (i) 10 min, (ii) 30 min and (iii) 1 hour for SWCNTs (**A**), DWCNTs (**B**), and MWCNTs (**C**).

A time dependant study taking samples at 10 minute intervals during 1064 nm laser irradiation at 260 mJ/pulse, and the VFD operating in the confined mode, was also undertaken for the three types of CNTs. Over time, more slicing and debundling was observed, with SWCNTs resulting in a narrower length distribution, but not so for DWCNTs and MWCNTs (Fig. 2), although here slightly higher yields of sliced CNTs were evident.

**Figure 3:**
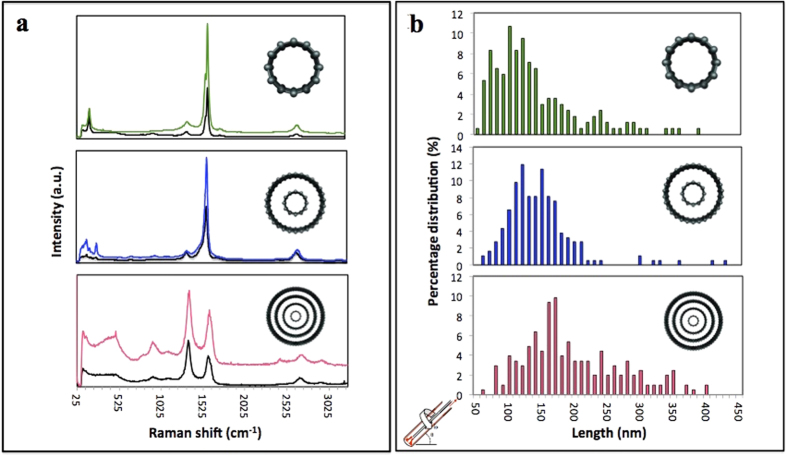
Raman spectroscopy and length distribution plots. (**a**) Raman spectra of SWCNTs, DWCNTs and MWCNTs, respectively, and the corresponding as received nanotubes (black). (**b**) Length distribution plots of the sliced SWCNTs, DWCNTs and MWCNTs under continuous flow operation of the VFD at a flow rate of 0.45 mL/min.

Experiments under confined mode established the potential for laser irradiation of CNTs in a VFD to produce unbundled, short CNTs, but for generating practical quantities of the materials, continuous flow processing is desirable. Here a jet feed of CNTs dispersed in a 1:1 mixture of water and NMP was directed into the bottom of the rapidly rotating 20 mm tube at a low flow rate of 0.45 mL/min, using the same concentration of CNTs as for the confined mode. The low flow rate was necessary for sufficient exposure time of the CNTs to the 8 mm diameter laser beam, while ensuring droplets of the suspension of the CNTs were striking the hemispherical bottom of the tube rather than a continuous stream of the liquid. Such droplets result in instability of the resulting thin film, which imparts additional shear compared to the confined mode of operation of the VFD *(24)*. Under continuous flow there is a dramatic increase in yield of the sliced CNTs, typically 95%, with a length distribution of 100–400 nm, for SWCNTs, DWCNTs and MWCNTs (Fig. 3(b)). Also noteworthy is that all the samples are devoid of long bundled nanotubes, unlike the outcome for confined mode of operation of the VFD.

### Raman analysis

Raman spectroscopy (Fig. 3(a)) established that any damage to the walls of the CNT products is minimal for confined mode and continuous flow operation of the VFD, irrespective of the number of shells of cylindrical graphene. Distinct peaks for CNTs include the radial breathing mode region (RBM), the tangential mode G band, its second harmonic G′ band and the disorder-induced D-band. The average ratio of intensities of the G and D bands (*I*_*D*_*/I*_*G*_) of the sliced SWCNT, DWCNT and MWCNT was ~0.2, ~0.3 and 1.2, respectively, which are similar to that of the starting pristine nanotubes which were ~0.2, ~0.2 and ~1.3, respectively, confirming that tube damage was minimal.

### Small angle neutron scattering analysis

The average size of the sliced CNTs in solution was analysed using small angle neutron scattering (SANS). The radius of SWCNTs treated with the laser alone and laser and VFD (confined mode, 10, 30 and 60 min) was 0.7 ± 0.2 nm, which is consistent with AFM results (0.7 to 1.4 nm). For shorter treatment times the nanotubes were longer, and in the absence of VFD processing there is either complete disruption of SWCNTs or no slicing, with the results consistent with the AFM data (supplementary data). Overall, SWCNTs irradiated and subject to shear are broken down to smaller length scales (Fig. S9–S12), as is the case for the DWCNTs (Fig. S13) and MWCNTs (Fig. S14), to 160 nm and 171 nm respectively (Table S1), which is again consistent with AFM data.

### Theoretical calculations

**Figure 4:**
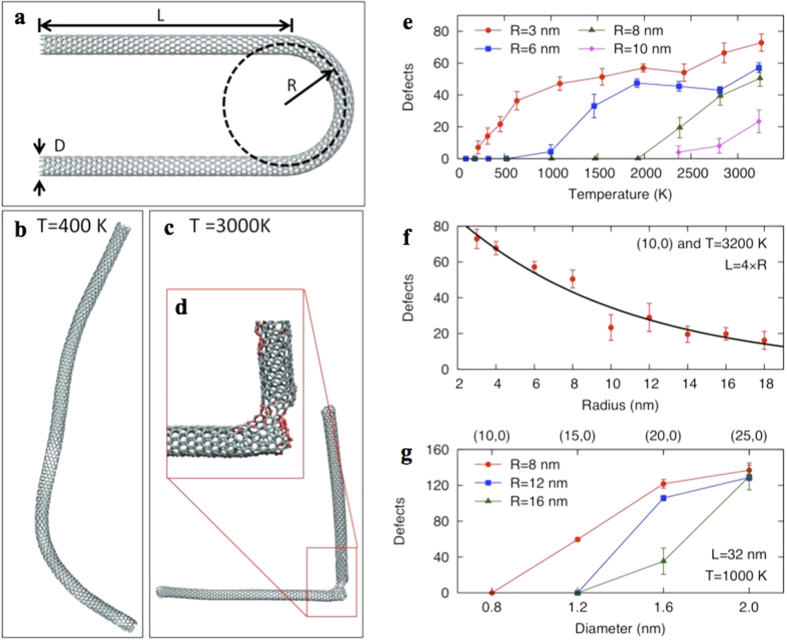
Theoretical calculations. (**a**) Initial bent structure of geometrical model of a nanotube showing the bending radius, R. and an arm-length, L, and nanotube diameter, D. (**b**,**c**) Snapshots of a (10, 0) nanotube model after annealing at 400 K and 3000 K respectively, with inset (**d**) showing a zoom-in of the defected sliced part of the nanotube. (**e**) Plot of the number of defects as a function of temperature for the (10, 0) nanotube with different bending radii. (**f**) Plot of the number of defects as a function of radius for the (10, 0) nanotube at a constant temperature of 3200 K. (**g**) Plot of number of defects at constant temperature as a function of diameter for different chiralities.

CNTs subjected to the shear forces created in the VFD result in local bending, as established by the observation that toroidal arrays of SWCNTs are produced in a mixture of toluene and water in the VFD in the absence of laser irradiation^33^. Bending is not surprising given the very high aspect ratio for SWCNTs, and when combined with heating from the laser it is likely to result in rupture of multiple bonds. To explore this further in understanding the mechanism of slicing, molecular dynamics simulations were carried out for SWCNTs, with hairpin-shaped tubes created to mimic the bending occurring in the VFD. Figure 4a shows a (10, 0) nanotube with a bending radius R of 2.5 nm and an arm-length L of 10 nm. When relaxed near room temperature (Fig. 4b and Movie S1), the hairpin unfolds and no defects are created. However, when the system is raised to a high temperature (Fig. 4c,d and Movie S2) a large tear occurs in the bent region and other defects appear nearby. The damage is produced by the extra energy provided by the elevated temperature resulting in breaking of bonds that are already strained.

The number of defects induced has been explored for different bending radii as a function of temperature. Figure 4e shows data for the same (10, 0) chirality with four bending radii, R = 3, 6, 8 and 10 nm. Ten simulations were performed at each temperature and radius to gather statistics. Defects are counted as atoms with a potential energy 10% higher than the cohesive energy of graphene or with a coordination number other than three. Below a critical temperature few defects are created, and in many instances no damage is created at all. As the radius increases, the critical temperature above which many defects are created becomes higher, as the bonds are less strained and require additional thermal activation to damage the nanotube. At large radii few defects are observed (Fig. 4f), even at high temperatures. These observations explain the experimental result that slicing occurs in the VFD with the laser irradiation but not with laser irradiation alone. Without the shear forces provided by the VFD, there is no localized bending or strained bonds for the laser to rupture. The simulations also suggest that the diameter D of the nanotube plays a role. Experimentally, this varies between 1 and 2 nm for the SWNTs. Figure 4g shows defect-vs-diameter data for three different bending radii. Chiralities of (10, 0), (15, 0), (20, 0) and (25, 0) were chosen with diameters spanning 0.78 to 2.0 nm. All three tubes were heated at 1000 K while bent in a hairpin-shape with radii of 8, 12 and 16 nm. For all three bending radii shown, the number of defects increases for nanotubes with larger diameter. Larger diameter tubes require less bending (i.e. larger bending radii) to be damaged. For example, while a (10, 0) nanotube heated at 1000 K needs to be bent to a radius less than 8 nm in order to show any defects, a (15, 0) nanotube shows damage at 8 nm but recovers for larger radii. This occurs due to the smaller inner bending radii (R-D/2) which arises for larger diameter tubes, while bonds at the outer bending radii are correspondingly stretched, with an outer radius of R + D/2. Therefore, tubes of larger diameter are more easily sliced. We expect that these simulations of SWCNTs are also applicable to DWCNTs and MWCNTs. Outer tubes will be damaged first, as they have a larger diameter. Once the outer tube of the MWCNT is breached then the mechanical force either side would provide a point for spontaneously slicing through the inner tubes.

In summary, we have established the ability to laterally slice carbon nanotubes within dynamic thin films, irrespective of the number of concentric layers in the material. This is without precedent, and further highlights the unique capabilities of the recently developed vortex fluidic device. Importantly, the method minimises the generation of defects on the CNTs, producing pristine material devoid of chemical stabilisers, and we have demonstrated that there is potential for scalability of the process under continuous flow mode of operation of the VFD. Controlling the lengths of the shorter nanotubes is more significant for SWCNTs, under confined mode, affording a narrow size distribution at a length scale suitable for drug delivery applications. In general, the availability of short single, double or multi-walled CNTs is poised for the advancement of their applications where specific length scale is paramount.

## Methods

### Preparation of aqueous suspensions of sliced CNTs

SWCNTs were purchased from Thomas Swan & Co (UK), as chemical vapour deposition prepared material with an as-received purity >90%, DWCNTs were purchased from Carbon Allotropes with an as received purity >99% and the MWCNTs were purchased from Sigma Aldrich with an as received purity >98%. Sample preparation included the addition of the CNTs (1 mg) into a sample vial containing a mixture of NMP and water (6 mL) at a 1:1 ratio. The solution mixture was then ultrasonicated for 10 minutes, affording a black stable dispersion. Under the confined mode operation, the solution mixture (1 mL) was then placed in the 20 mm borosilicate NMR glass tube (ID 16.000 ± 0.013 mm) and placed in the vortex fluidic device (VFD), under the optimized rotating speed of 7500 rpm, at a tilt angle of 45 degrees. Simultaneously, a nanosecond pulsed laser processing system with an energy of approximately 260 mJ was applied to the rapidly rotating system for a period of time (10 minutes, 30 minutes and 1 hour), depending on the resulting length distribution. Under continuous flow mode, jet feeds with a flow rate at 0.45 mL/min (optimised) deliver the CNT suspension (mixture CNTs in NMP/water at a 1:1 ratio-similar concentration, as for the confined mode) into the rapidly rotating tube. Centrifugation (g 3.22) of the resulting solution for the confined mode of operation was required to remove any large agglomerates, unsliced bundled CNTs and impurities in the sample. The centrifugation step was not necessary for the continuous flow mode of operation with *ca* 95% of the material isolated as sliced CNTs.

### Computational details

Starting coordinates were obtained by geometrically mapping the coordinates of a straight carbon nanotube onto a semicircle of a given radius linked with two straight sections to give a U-shape. Molecular Dynamics (MD) simulations were performed using the Environmental-Dependent Interaction Potential (EDIP) for carbon^37^. Prior to the MD step, the structures were relaxed using steepest descent optimization to remove any forces associated with the mapping. All MD simulations were performed in the NVE (constant particle-volume-energy) ensemble with an integration timestep of 0.35 fs. The simulations were initialized with velocities drawn from a Maxwell-Boltzmann distribution at a prescribed temperature and the entire system was allowed to evolve for 35 ps. Due to equipartition, the system temperature during the simulation was very close to half the initial value. Note that all temperatures reported here are system averages, and not the starting value. Steepest descent optimization was again applied to the final structures to remove any displacements due to thermal vibrations. The resultant structure was then analysed for defects. Atoms were defined as defects if their coordination number (defined by Equation 9 in Ref. 37) differed by more than 0.1 from the perfect value of 3 or if potential energy was more than 10% less than the perfect value of −7.361 eV for graphite.

### Instrumentation

TEM characterizations were carried out using the Phillips CM 200 operating at 200 kV, in the Adelaide Microscopy at the University of Adelaide. SEM analysis was carried out on the FEI Quanta 450 in Adelaide Microscopy. AFM, Raman characterizations and UV-Vis analysis were performed at Flinders University. SANS characterizations were carried on QUOKKA at the Bragg Institute, ANSTO.

## Additional Information

**How to cite this article**: Vimalanathan, K. *et al.* Fluid dynamic lateral slicing of high tensile strength carbon nanotubes. *Sci. Rep.* **6**, 22865; doi: 10.1038/srep22865 (2016).

## Supplementary Material

Supplementary Information

Supplementary Movie S1

Supplementary Movie S2
