# Supplementary material for: Fluid dynamic lateral slicing of high tensile strength carbon nanotubes
Source: Sci Rep. 2016 Mar 11;6:22865. doi: 10.1038/srep22865 (PMC4786806; doi:10.1038/srep22865)
Supplement: Supplementary Information [file srep22865-s1.pdf]

Supplementary Information

Fluid dynamic slicing of super tensile carbon nanotubes

Kasturi Vimalanathan,[1] Jason R. Gascooke,[1] Irene Suarez-Martinez,[2] Nigel Marks,[2] Harshita Kumari,[3,4] Chris Garvey,[5] Jerry L. Atwood,[3] Warren D. Lawrance,[1] and Colin L. Raston[1*]

1 Flinders Centre for NanoScale Science & Technology, School of Chemical & Physical Sciences, Flinders University, Adelaide SA 5001, Australia

2 Nanochemistry Research Institute, Department of Physics and Astronomy, School of Science, Curtin University, Bentley Campus, Perth, WA 6102, Australia

3 Department of Chemistry, University of Missouri, 601 South College Avenue, Columbia, Missouri 65211, United States

4 James L. Winkle College of Pharmacy, University of Cincinnati, 3225 Eden Avenue, Cincinnati, Ohio, 42567, United States

5 Bragg Institute, Australian Nuclear Science and Technology Organisation, New Illawarra Road, Lucas Heights, NSW 2234

*Corresponding authors email: colin.raston@flinders.edu.au

Raman characterization

In a typical carbon nanotube Raman spectrum[1-3], the RBM frequencies correspond to the diameter of the nanotubes present in the sample, correlating to peak intensities within the specified region. Any disorder induced D-band conforms to damage/defects present within the walls/ends of individual CNTs, with the ratio of intensity of the G band and the disordered induced D band (I_D/I_G) determining the structural defects present on the walls of the nanotubes post-processing.

Supplementary Figure 1

AFM images of the as received SWCNTs, MWCNTs and DWCNTs respectively (in the absence of the VFD and laser) dispersed in a 1:1 mixture of NMP and water after batch processing in a round bottom flask (stirring).

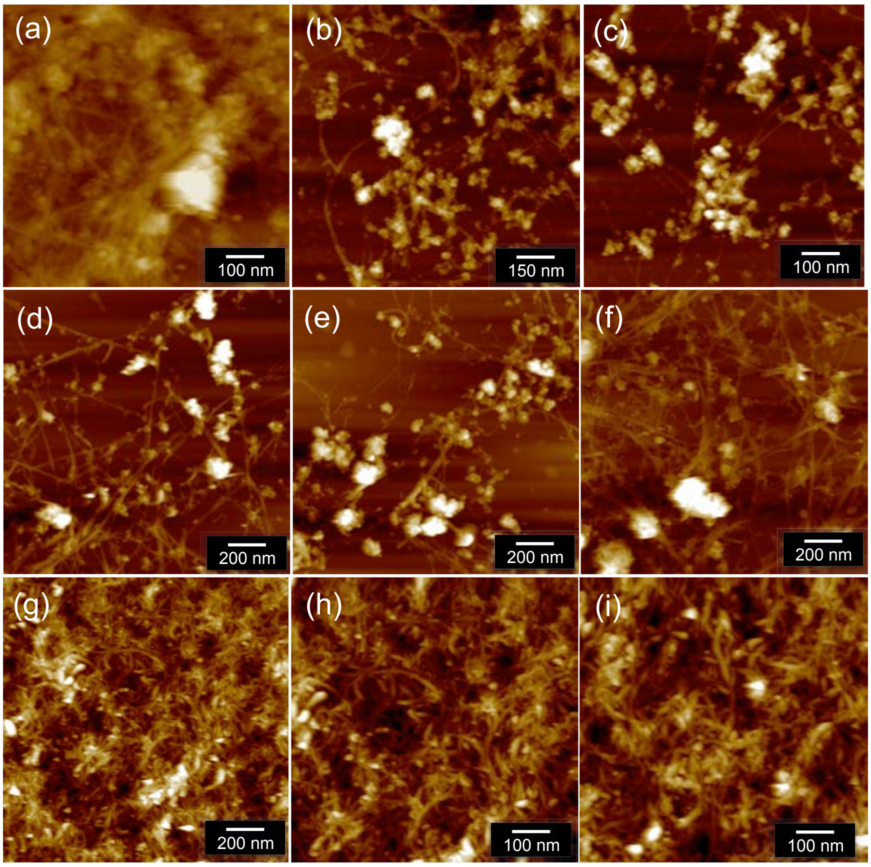

Fig. S1: AFM images of the as-received carbon nanotubes (a-c) SWCNTs (d-f) DWCNTs, and (g-i) DWCNTs.

Supplementary Figure 2,3 and 4

Additional AFM images of the sliced carbon nanotubes, SWCNTs, DWCNTs and MWCNT.

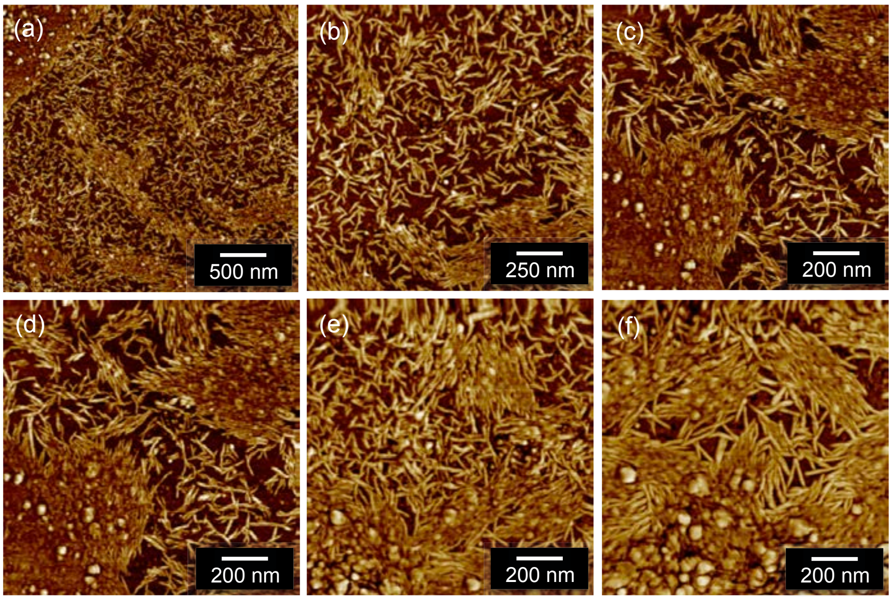

Fig. S2: AFM images of the sliced SWCNTs.

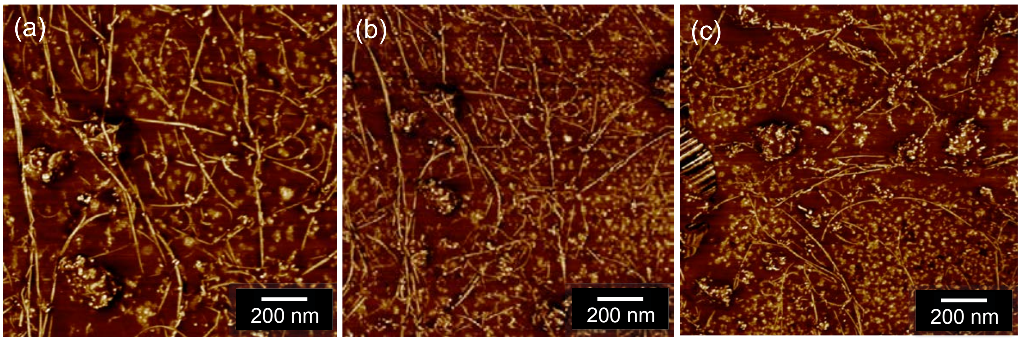

Fig. S3: AFM images of the sliced DWCNTs.

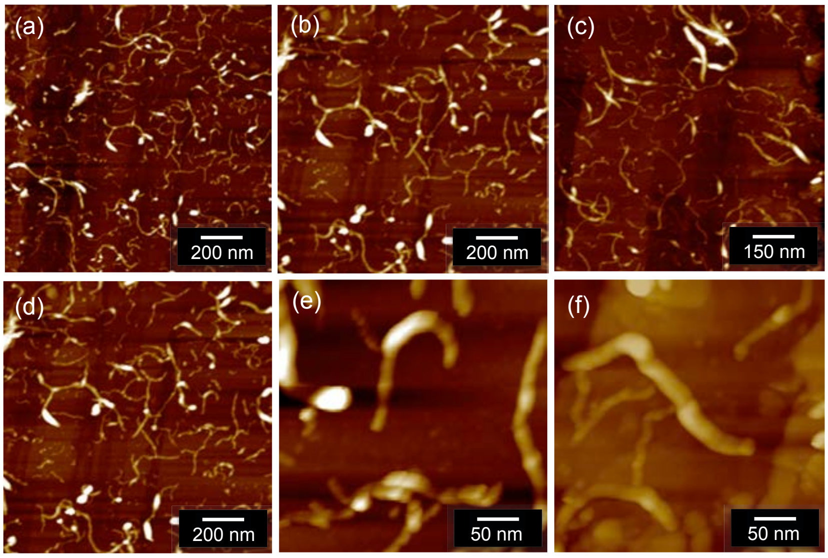

Fig. S4: AFM images of the sliced MWCNTs .

Supplementary Figure 5

AFM images SWCNTs, MWCNTs and DWCNTs respectively processed with the 1064 nm laser (in the absence of VFD processing) for a 30 minute processing time. A glass cuvette was filled with the respective CNT suspension (1:1 mixture of NMP and water) with a stirring bar beneath the vial to ensure continuous mixing of the suspension. The 8 mm pulsed laser was directed to the middle of the glass cuvette.

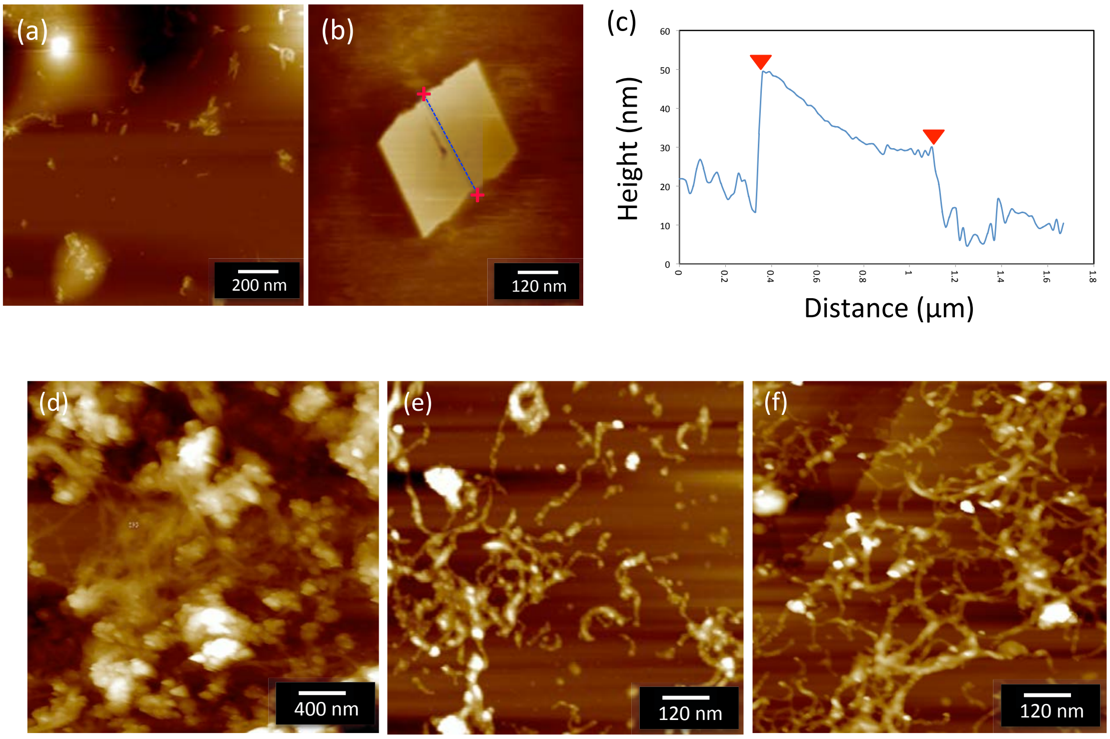

Fig. S5: (a) AFM height images of the fragmented SWCNTs, (b) AFM height image of a SWCNT fragment and its (c) associated height profile, (d) AFM height image of DWCNTs, and (e-f) AFM height images of partially sliced MWCNTS with no uniformity in length with the presence of bundles and agglomerates.

Supplementary Figure 6

Control experiments to study the effects of changing the laser wavelength on the slicing of the CNTs at the optimised laser power, 260 mJ. The 532 nm wavelength (green light) showed no apparent slicing of the CNTs, single, doubled and multi walled under the same conditions.

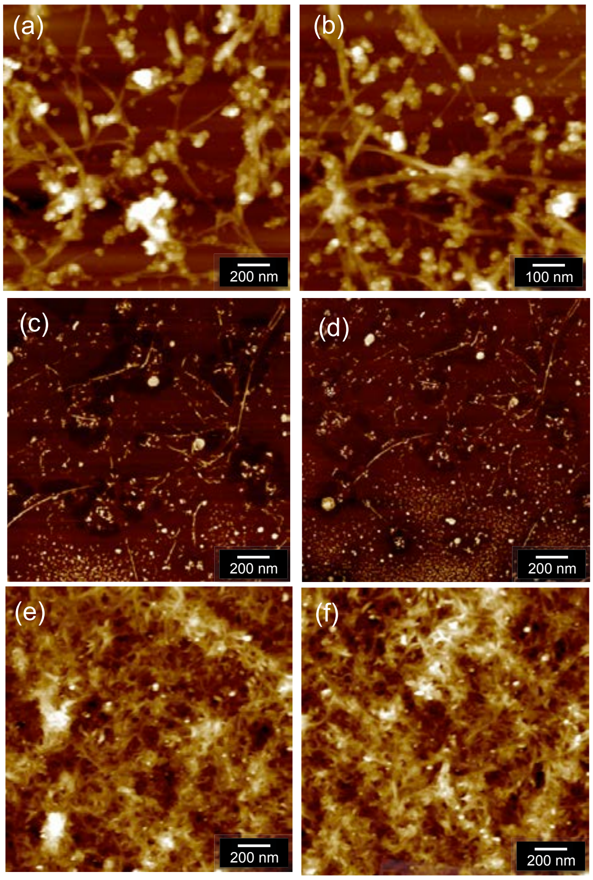

Fig. S6: AFM images of the nanotubes; (a-b) SWCNT, (c-d) DWCNT, and (e-f) MWCNT.

Supplementary Figure 7

Control experiments to establish the optimised power for the slicing process. At a laser power of 150 mJ, bundled SWCNT remained unaffected with debundling and some agglomerates still present, however no slicing was evident. At 450 mJ, there were large amounts of bundled and agglomerated SWCNTs still present within the sample; however, small amounts of sliced SWCNTs were observed (Figure (e)) albeit irregular in length.

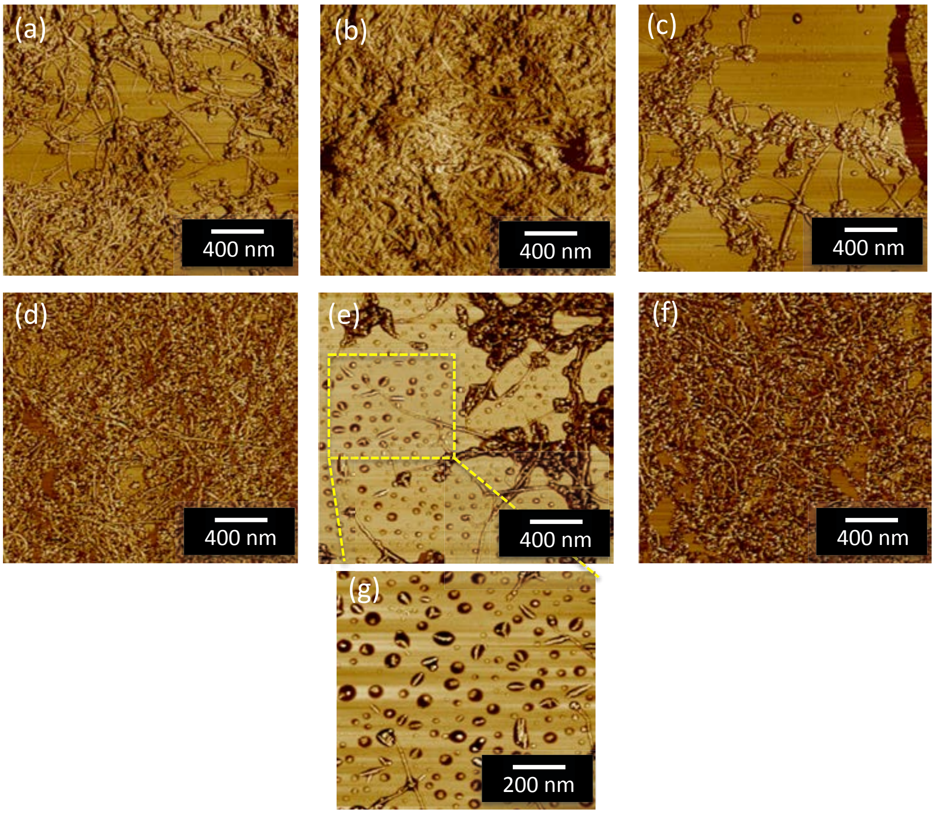

Fig. S7: AFM images of SWCNT processed in the VFD (θ 45°, rotational speed 7500 rpm) at different laser powers for a 10 minute, 30 minute and 1 hour processing time respectively (VFD confined mode); (a-c) 150 mJ (a) 10 minutes, (b) 30 minutes, (c) 1 hour; (d-f) 450 mJ (d) 10 minutes, (e,g) 30 minutes- small amounts of sliced SWCNTs were observed, and (f) 1 hour.

Supplementary Figure 8

Absorption spectrums of the sliced single, double and multi CNTs were observed to determine the concentration and their chiralities, present in the aqueous dispersions under both confined mode (Supplementary Fig. 6(a)) and continuous flow (Supplementary Fig. 6(b)). For SWNCTs there are four regions around 850-1350 nm, 500-850 nm, 330-450 nm and 300-400 nm, corresponding to the S_{11}, S_{22}, S_{33} and S_{44} optical transition of the semiconducting SWCNTs. The absorbance peaks observed between the regions 400-650 nm represent the first optical transition of metallic SWCNTs (M_{11}). As for the sliced CNTs for DWCNTs and MWCNTs, the absorption peaks were significantly broader compared to the peaks for SWCNTs. A much lower absorbance was observed specifically for the MWCNTs. The absorption peaks for DWCNTs and MWCNTs are in similar regions to the SWCNTs. The peaks between 950 and 1250 nm correspond to small amounts of semiconducting nanotubes for the S_{11} transition and dominated amounts of the S_{22} transition, corresponding to the smaller diameter inner wall nanotubes and the larger outer walls respectively. A typical absorption peak at 273 nm corresponds to the surface π-plasmon excitation of stable dispersed carbon nanotube dispersions, also known as the ultraviolet absorption characteristic of the nanotubes[4-5].

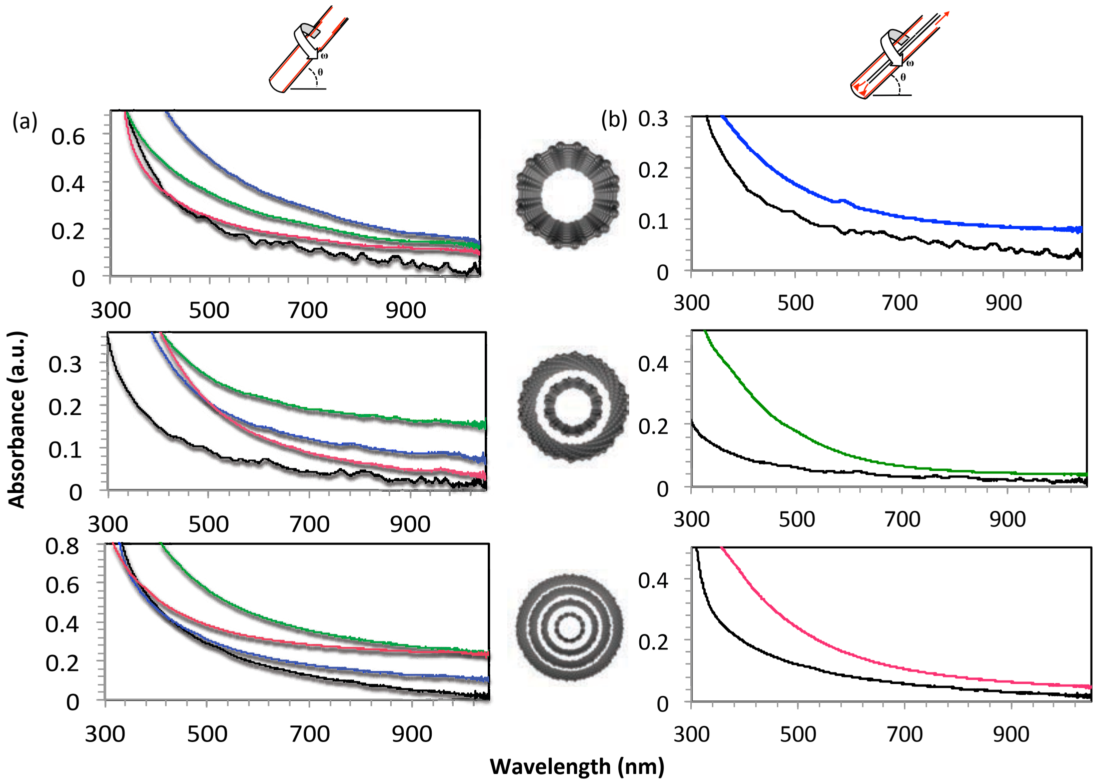

Fig. S8: UV-Visible spectras of the sliced SWCNT, DWCNT and MWCNT compared to the respective as-received CNTs; (a) under confined mode, (b) under continuous flow

Supplementary Figure 9-14

SANS measurement was performed along with AFM to compare the results from the two techniques and in two different solvents. While AFM provides 2-dimensional real space representation of structure with some limited statistics, SANS as a reciprocal space method where 3-dimensional real space information is reconstructed by modeling of scattering curves[6], provides a true statistical perspective on structure. The two techniques are therefore complementary. For this purpose, we evaporated NMP from sliced/pre-treated (shear+laser or laser only) nanotubes. The resultant material/nanotubes were re-dissolved in deuterated chloroform for SANS measurements. Deuterated solvent was used to lower the relative incoherent background scattering and the contrast between the solute and the solvent. SANS measurements were performed on QUOKKA at Braggs Institute, ANSTO. Neutrons of wavelength 5 Å were used with a wavelength spread/ full-width half maximum of $\Delta\lambda/\lambda$ = **12%**. The sample to detector distances of 1.3 m, 4.5 m, 13 m covered the overall q range of 0.013 Å$^{-1} < q < 0.534$ Å^{-1}. Here $q = (4\pi/\lambda) \sin (\theta/2)$ is the magnitude of the scattering vector and θ is the scattering angle. The sample scattering was corrected for the background, the empty cell scattering, and the sensitivity of the individual detector pixels. The corrected data sets were placed on an absolute scale and the structure was modeled using Igor Pro software[7].

Small-angle neutron scattering (SANS) data for single-walled-, double walled- and multi-walled carbon nanotubes, subjected to laser and shear at various time scales, have been reduced and fitted to cylindrical models. The quality of the fit and physical plausibility of the structural parameters was used to evaluate which structure was the most likely solution structure of the nanotubes. The scattering length densities (SLDs) for the nanotubes and solvent (*d3*-chloroform) were calculated and the analyses were done on Igor software provided by NIST. The data was collected at Bragg Institute, ANSTO. The data was fitted to cylinder model. In the smeared cylindrical fits (all 3 types), the SLDs for SWCNTs/ DWCNTs/ MWCNTs were free, whereas that for the solvent d_3-$CDCl_3$ was held fixed at the calculated values and the structural parameters common to each data set were optimized to provide the best fit. The scattering data indicates that vortex fluidic device (VFD) in combination with laser enabled the breaking of carbon nanotubes. A list of fits and individual fits with fitting parameters and notes are enlisted below.
In the smeared cylindrical fits, the scattering length densities/SLDs for the solvent d_3-$CDCl_3$ and length were held fixed at the calculated values and the structural parameters common to each data set were optimized to provide the best fit.

TABLE 1: RESULT SUMMARY

Sample	SLD	Radius	Length
SWCNT laser only	5.91621e-06 ±0.000724898	7 ± 0.502443	999.523±283.901
SWCNT laser +shear (10min)	4.71192e-06 ± 0.000482733	7 ± 2.2327	3700 ±0
SWCNT laser +shear (30min)	5.71086e-06 ± 0.000462135	7 ± 1.75552	1650 ±0
SWCNT laser +shear (60min)	5.9024e-06 ± 0.000740086	7 ± 2.6338	1100 ±0
DWCNT laser +shear(60min)	4.86312e-06 ± 0.00244807	20 ± 1.22684	1600 ±0
MWCNT laser +shear(60min)	4.42184e-06 ± 0.00369752	45 ± 1.72077	1710 ±0

Fit to SmearedCylinderForm,
Data file: a1_comb_ABS

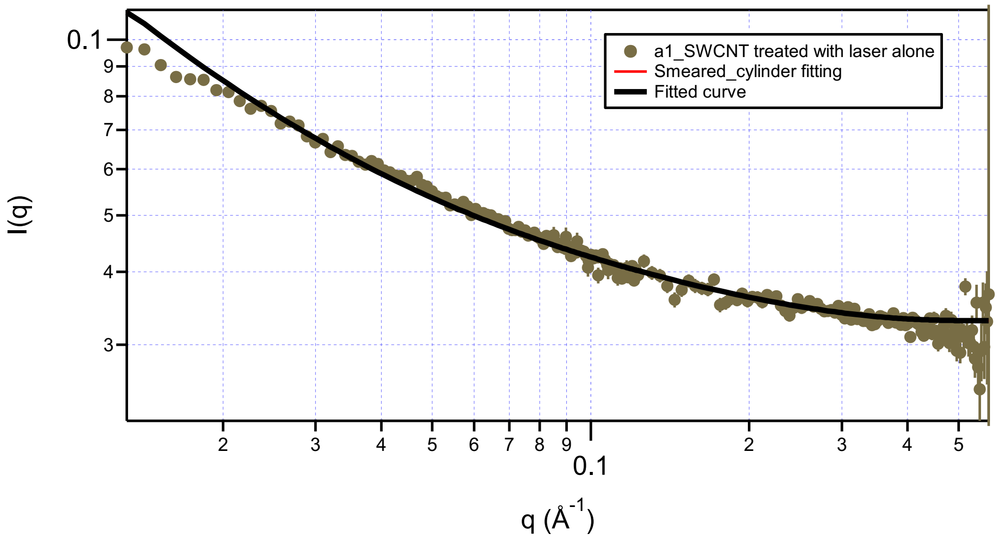

scale	0.00290988	±	1.5284
radius (Å)	7 ±	0.502443	
length (Å)	999.523	±	283.901
SLD cylinder (Å^{-2})	5.91621e-06	±	0.000724898
SLD solvent (Å^{-2})	3.156e-06	±	0
incoh. bkg (cm^{-1})	0.032984	±	0.000181443

chisq = 693.141
Npnts = 223 Sqrt(χ^2/N) = 1.76302
Fitted range = [0,222] = 0.0131333 < Q < 0.570667
FitError = No Error FitQuitReason = No decrease in chi-squared

Fig. S9: Smeared cylinder fitting for single-walled carbon nanotubes treated with laser only

Fit to SmearedCylinderForm,
Data file: a2_comb_ABS

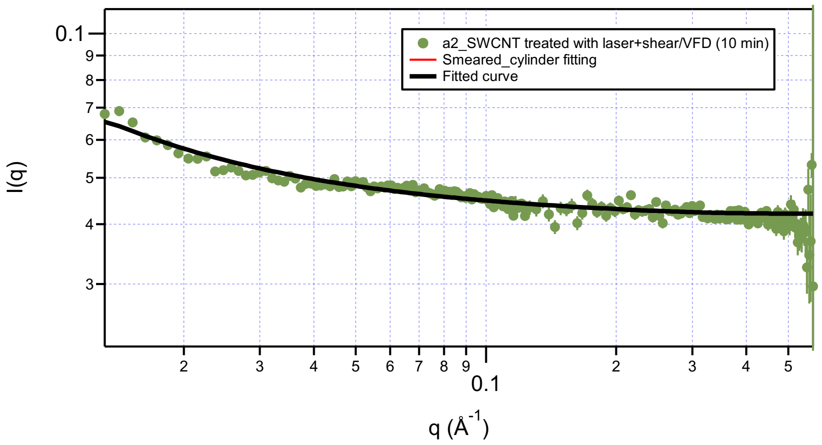

scale	0.00264772	±	1.64283
radius (Å)	7 ±	2.2327	
length (Å)	3700 ±	0	
SLD cylinder (Å^{-2})	4.71192e-06	±	0.000482733
SLD solvent (Å^{-2})	3.156e-06	±	0
incoh. bkg (cm^{-1})	0.0420775	±	0.000234206

chisq = 441.902
Npnts = 216 Sqrt(χ^2/N) = 1.43033
Fitted range = [0,215] = 0.0131333 < Q < 0.570667
FitError = No Error FitQuitReason = No Error

Fig. S10: Smeared hollow cylinder fitting for single-walled carbon nanotubes treated with laser and shear 10 minutes

Fit to SmearedCylinderForm,
Data file: a3_comb_ABS

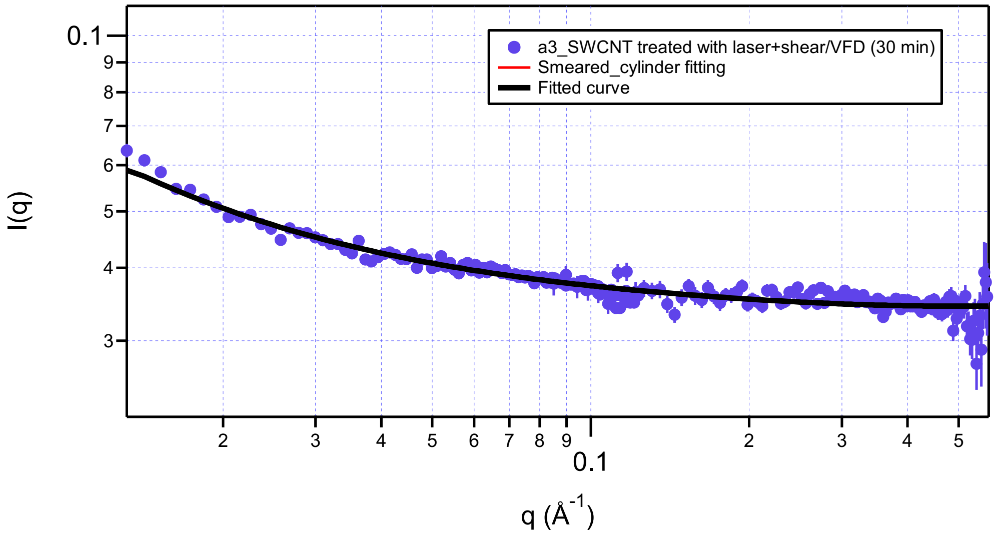

scale	0.00104287	±	0.377255
radius (Å)	7 ±	1.75552	
length (Å)	1650 ±	0	
SLD cylinder (Å^{-2})	5.71086e-06	±	0.000462135
SLD solvent (Å^{-2})	3.156e-06	±	0
incoh. bkg (cm^{-1})	0.0343739	±	0.000195543

chisq = 337.927
Npnts = 223 Sqrt(χ ^2/N) = 1.231
Fitted range = [0,222] = 0.0131333 < Q < 0.570667
FitError = No Error FitQuitReason = No Error

Fig. S11: Smeared hollow cylinder fitting for single-walled carbon nanotubes treated with laser and shear 30 minutes

Fit to SmearedCylinderForm,
Data file: a4_comb_ABS

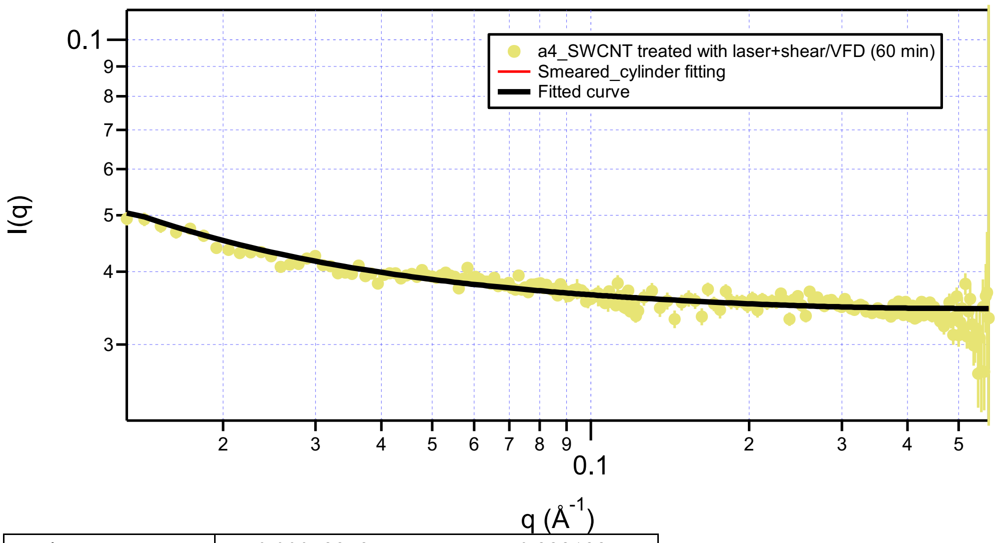

scale	0.00059959	±	0.323132
radius (Å)	7 ±	2.6338	
length (Å)	1100 ±	0	
SLD cylinder ($Å^{-2}$)	5.9024e-06	±	0.000740086
SLD solvent ($Å^{-2}$)	3.156e-06	±	0
incoh. bkg (cm^{-1})	0.0345868	±	0.000193535

chisq = 372.465
Npnts = 222 Sqrt(χ ^2/N) = 1.29529
Fitted range = [0,221] = 0.0131333 < Q < 0.570667
FitError = No Error FitQuitReason = No Error

Fig. S12: Smeared hollow cylinder fitting for single-walled carbon nanotubes treated with laser and shear 60 minutes

Fit to SmearedCylinderForm,
Data file: a5_comb_ABS

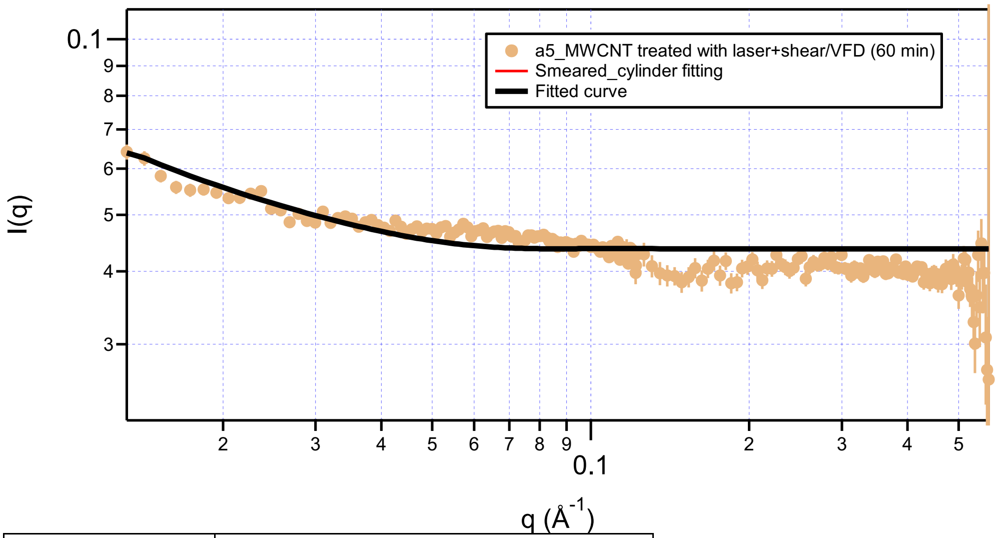

scale	9.24342e-05	±	0.540001
radius (Å)	45 ±	1.72077	
length (Å)	1710 ±	0	
SLD cylinder (Å^{-2})	4.42184e-06	±	0.00369752
SLD solvent (Å^{-2})	3.156e-06	±	0
incoh. bkg (cm^{-1})	0.0437142	±	6.81163e-05

chisq = 1616.97
Npnts = 216 Sqrt(χ ^2/N) = 2.73605
Fitted range = [0,215] = 0.0131333 < Q < 0.570667
FitError = No Error FitQuitReason = No Error

Fig. S13: Smeared hollow cylinder fitting for multi-walled carbon nanotubes treated with laser and shear 60 minutes

Fit to SmearedCylinderForm,
Data file: a6_comb_ABS

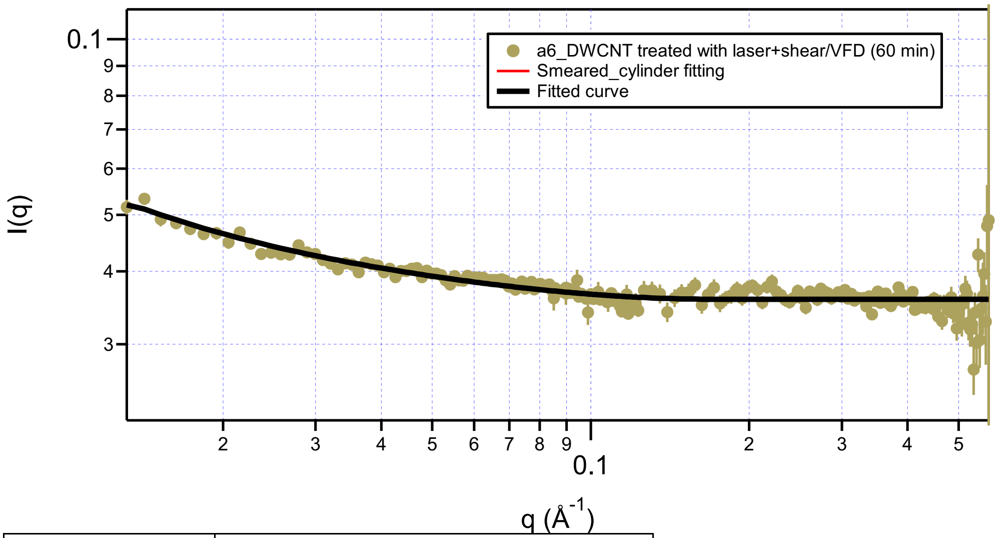

scale	0.000193797	±	0.555824
radius (Å)	20 ±	1.22684	
length (Å)	1600 ±	0	
SLD cylinder (Å^{-2})	4.86312e-06	±	0.00244807
SLD solvent (Å^{-2})	3.156e-06	±	0
incoh. bkg (cm^{-1})	0.0357999	±	9.45695e-05

chisq = 316.69
Npnts = 223 Sqrt(χ ^2/N) = 1.19169
Fitted range = [0,222] = 0.0131333 < Q < 0.570667
FitError = No Error FitQuitReason = No Error

Fig. S14: Smeared hollow cylinder fitting for double-walled carbon nanotubes treated with laser and shear 60 minutes

Supplementary Videos

Movie S1: Relaxation at 400K for a (10,0) nanotube, where the hairpin unfolds and no defects are created.

Movie S2: Relaxation at 3000K for a (10,0) nanotube, where a large tear occurs in the bent region and other defects appear nearby.

References

1. Dresselhaus, M.S., Dresselhaus, G., Saito, R. & Jorio, A. Raman spectroscopy of carbon nanotubes. *Phys. Rep.* **409**, 47-99 (2005).
2. Hennrich, F. et al. Raman spectroscopy of individual single walled carbon nanotubes from various sources. *J.Phys. Chem. B.* **109**, 10567-10573 (2005).
3. Bokobza, L. & Zhang, J. Raman spectroscopy characterization of multiwall carbon nanotubes and of composites. *Poly. Lett.* **6**, 601-608 (2012).
4. Hubble, L.J., Clark, T.E., Makha, M. & Raston, C.L. Selective diameter uptake of single-walled carbon nanotubes in water using phosphonated calixarenes and 'extended arm' sulfonated calixarenes. *J. Mater. Chem.* **18**, 5961-5966 (2008).
5. Attal, S., Thiruvengatham, R. & Regev, O. Determination of the concentration of SWCNTs in aqueous dispersions using UV-Vis absorption spectroscopy. *Anal. Chem.* **78**, 8098-8104 (2006).
6. Pedersen, J.S. Analysis of small-angle scattering data from colloids and polymer solutions: modelling and least squares fitting. *Adv. Colloid Interface Sci.* **70,** 171-210 (1997).
7. Kline, S. R. Reduction and analysis of SANS and USANS data using IGOR Pro. *J., Appl. Crystallogr.* **39**, 895 – 900 (2006).
